# OMANISHA: A benchmark dataset for identifying and categorizing Bengali misogynistic text

**DOI:** 10.1016/j.dib.2026.113301

**Published:** 2026-10-03

**Authors:** Fatama Jannat Tisha, Bibhas Roy Chowdhury Piyas, Nurjahan Afrose, Sabbir Ahmed, Shahrin Islam, Shazzad Hossen, Jafrin Iqbal Chowdhury, Afsana Begum, Imran Mahmud

**Affiliations:** a Department of Software Engineering, Daffodil International University, Dhaka, 1216, Bangladesh; b Department of Computer Science and Engineering, Daffodil International University, Dhaka, 1216, Bangladesh

**Keywords:** Bengali misogynistic text detection, Online misogyny, Online abuse detection, Natural language processing, Hate speech detection, Gender-based online violence, Low-resource language, Social media analysis

## Abstract

Misogynistic discourse in online spaces has profound psychological, social, and institutional consequences for women, contributing to persistent gender inequalities, the normalization of gender-based violence, and the suppression of female voices in digital communities. The unchecked proliferation of such hostile content across social media platforms has made automated misogyny detection a pressing social imperative. Despite Bengali’s global prominence, computational resources for detecting gender-based online abuse remain limited. To address this gap, we introduce OMANISHA (**O**nline **M**isogynistic **A**nnotated **N**atural-language **I**nstances for **S**entiment and **H**ate **A**nalysis**),** a Bengali dataset designed to advance misogynistic text detection. The dataset comprises 7,017 annotated samples independently developed by the authors based on linguistic patterns observed across diverse public online platforms. Each sample was assigned to one of four predefined labels: non-misogynistic (2,420), stereotype (1,744), derogation (1,527), and sexual harassment (1,326). Moreover, English translations are provided to enhance cross-lingual accessibility. Every sample was independently labeled by two native Bengali-speaking annotators, chosen from a four-member annotation team representing diverse gender, religious, ethnic, and geographical backgrounds. Annotation disagreements were resolved through structured consultation with a third annotator. Annotation quality was assessed using Cohen’s Kappa (κ = 0.76) and Krippendorff’s Alpha (α = 0.75), indicating substantial inter-annotator agreement and strong annotation reliability. Pairwise Jaccard Similarity scores among the classes range from 0.12 to 0.21, suggesting taxonomic distinctiveness across the defined categories. The preprocessing stages include duplicate removal, text normalization, and coherence checking to ensure overall data integrity. OMANISHA serves as a publicly accessible dataset for supporting explainable AI-driven content moderation, advancing low-resource NLP research, and promoting women-centric online safety.

Specifications Table

**Table:** 

Subject	Computer Science
Specific subject area	Natural Language Processing, Machine Learning, Bengali Misogynistic Text Identification, Text Classification
Type of data	Text Files (xlsx-formatted)
Data collection	The 7,017 Bengali text samples were independently developed following an exploratory review of naturally occurring Bengali online discourse. The review identified recurring linguistic and contextual patterns, including supportive statements, gender stereotypes, derogatory expressions, morality-based criticism, sexualized remarks, objectification, and gender-directed hostility. These real-world patterns and interaction contexts guided the generation and annotation of samples into four classes: Non-misogynistic, Stereotype, Derogation, and Sexual Harassment. No original online post or comment was copied or directly paraphrased. Two annotators, selected from a pool of four individuals familiar with Bengali linguistic conventions and socio-cultural contexts, independently labelled each sample. Any disagreements were resolved by a third annotator, while additional validation by native Bengali speakers further strengthened the consistency and reliability of the corpus for Bengali NLP research.
Data source location	The linguistic and contextual patterns used to guide dataset development were observed in publicly accessible Bengali online discourse across Facebook public pages, posts, discussions and reels, YouTube comments, Instagram posts and reels, Twitter/X discussions, online news portals and comment sections, and selected public blogs and forums. Particular attention was given to discussions on women’s achievements, employment, leadership, relationships, marriage, clothing and appearance, entertainment, violence against women, and sexual harassment, where recurring misogynistic and non-misogynistic patterns were identified.
Data accessibility	Repository name: Mendeley Data Data identification number: doi:10.17632/87767vkp84.3 Direct URL to data: https://data.mendeley.com/datasets/87767vkp84/3
Related research article	None

## 1 Value of the Data

• OMANISHA is the first publicly available, large-scale annotated Bengali dataset explicitly developed for misogynistic text identification, with mutually exclusive classes covering non-misogynistic, stereotype, derogation, and sexual harassment. It fills a significant gap in the resources available, and serves as a benchmark for the development of Bengali NLP in the future.
• This dataset serves as a valuable linguistic resource for researchers, developers, and platform safety teams to create, train, and thoroughly test automated models for detecting misogyny in the context of Bengali-speaking online communities.
• OMANISHA allows for a fine-grained classification of different types of misogynistic expressions, which can help to develop more precise and context-sensitive content moderation systems, unlike binary hate speech datasets that lump nuanced forms of gendered hostility into a single harmful category.
• The dataset captures both implicit and explicit toxicity towards women in various linguistic patterns, allowing for robust and context-aware detection of harmful content targeting women, thereby facilitating more effective automated content moderation for digital safety.
• The dataset is aligned with UN SDG 5 – Gender Equality, and it contributes to promoting women's safe and inclusive spaces online. It invites future researchers, linguists, and platform safety practitioners to continue future research on women-centric online safety and gendered aggression in low-resource language settings.
• OMANISHA serves as a valuable benchmark for developing and evaluating modern NLP architectures, including BERT-based models, multilingual transformers, and large language models (LLMs). Its fine-grained annotations enable practitioners to fine-tune and compare these models for multiclass Bengali misogyny detection, providing a practical benchmark for assessing their ability to identify both explicit and implicit forms of gendered abuse in a low-resource language setting.

## 2 Background

With the proliferation of social media, toxic, abusive, and misogynistic information is spreading at a rapid pace, posing a significant challenge to online safety and automated content moderation. In response, researchers have created many datasets and computational techniques to aid in the identification and classification of harmful online speech.

Guest et al. (2017) [1] developed a Reddit-based corpus of 6,567 expert-annotated posts and comments, along with a hierarchical taxonomy that represents the various ways in which misogynistic expression occurs. Taking this research one step further to multimodal content, Gasparini et al. [2] created a benchmark dataset of 800 misogynistic and non-misogynistic memes, allowing for the study of cybersexism through both textual and visual information. Further, Hashmi et al. [3] explored machine learning, deep learning, transformer-based, and explainable artificial intelligence methods, showcasing their ability to identify and comprehend gender-based abusive content across online platforms.

The recent rise in the demand for harmful content detection in low-resource languages has also motivated the creation of a few Bengali NLP resources. ToxLex [4] is a large-scale Bangla toxic-language dataset collected from Facebook comments that contains various types of toxic language including misogynistic and hate-related language. To include conversational information, Rupa et al. [5] created a context-aware Bangla toxic-comment dataset that contains the surrounding interactions with a particular comment. Beyond written text, Hossain et al. [6] presented a Bengali abusive-speech corpus consisting of 6,100 audio clips for speech offensive-language detection.

There has also been a growing focus on the detection of Bengali cyber bullying and abusive content. Jahin et al. [7] used transformer-based architectures on a large scale social media dataset and showed their effectiveness in the detection of harmful online interactions. A similar study [8] used machine learning and explainable AI methods to enhance the interpretability of the model and its ability to classify abusive content.

Other types of online aggression and affective language have also been investigated in Bengali. Rashid et al. [9] created ALERT, a Bengali corpus which consists of 4027 labeled instances of religiously aggressive content with four classes: vandalism, hate speech, no aggression and atrocity. Based on this resource, the study in [10] proposed a transformer-based framework using a modified attention mechanism and an ensemble-learning approach to enhance the classification performance. In another domain, Faisal et al. [11] created a Bengali and Banglish emotion dataset for fine-grained emotion classification for low resource language research.

While these studies have made significant contributions to the field of Bengali toxicity detection, abusive language detection, religious aggression detection, emotion detection, and cyberbullying detection, research efforts dedicated to multiclass misogyny detection are still limited. To address this gap, OMANISHA introduces an annotated Bengali dataset with English translations for multiclass misogyny classification.

## 3 Data Description

OMANISHA was developed to capture diverse forms of misogynistic language in Bengali online discourse. There are a total of 4 mutually exclusive class labels for each instance. Each category was carefully formulated with clear conceptual boundaries in order to make the annotation clear and consistent.

1. **Non-Misogynistic:** Texts that do not contain misogynistic, discriminatory, abusive, or hostile expressions toward women or girls. These instances reflect neutral, positive, respectful, or supportive content related to women’s education, career development, leadership, independence, family support, rights, and gender equality, without reinforcing prejudice, stereotypes, or gender-based hostility.
2. **Stereotype:** Texts that reinforce generalized beliefs, assumptions, or socially prescribed expectations about women based on gender. This category includes expressions reflecting patriarchal or traditional views regarding women’s roles, behavior, morality, competence, autonomy, or social responsibilities. Such texts may promote expectations related to obedience, marriage, domesticity, modesty, or restricted independence and typically reflect collective judgments about women as a group rather than direct attacks on a specific individual.
3. **Derogation:** Texts that demean, insult, or belittle a specific woman or girl by attacking personal, social, intellectual, professional, or physical attributes. These expressions commonly target appearance, intelligence, education, occupation, lifestyle, confidence, or social standing and are intended or likely to humiliate, ridicule, or express contempt.
4. **Sexual Harassment:** Texts that include unwanted sexual remarks, sexual objectification, sexually suggestive expressions, or inappropriate sexual advances toward women or girls. These instances often involve vulgar or degrading references to appearance, body parts, or sexual characteristics, with the intent of harassment, objectification, humiliation, or sexualized degradation.

The OMANISHA dataset was created through a systematic data development process, which is summarized in Fig. 1.

**Fig. 1:**
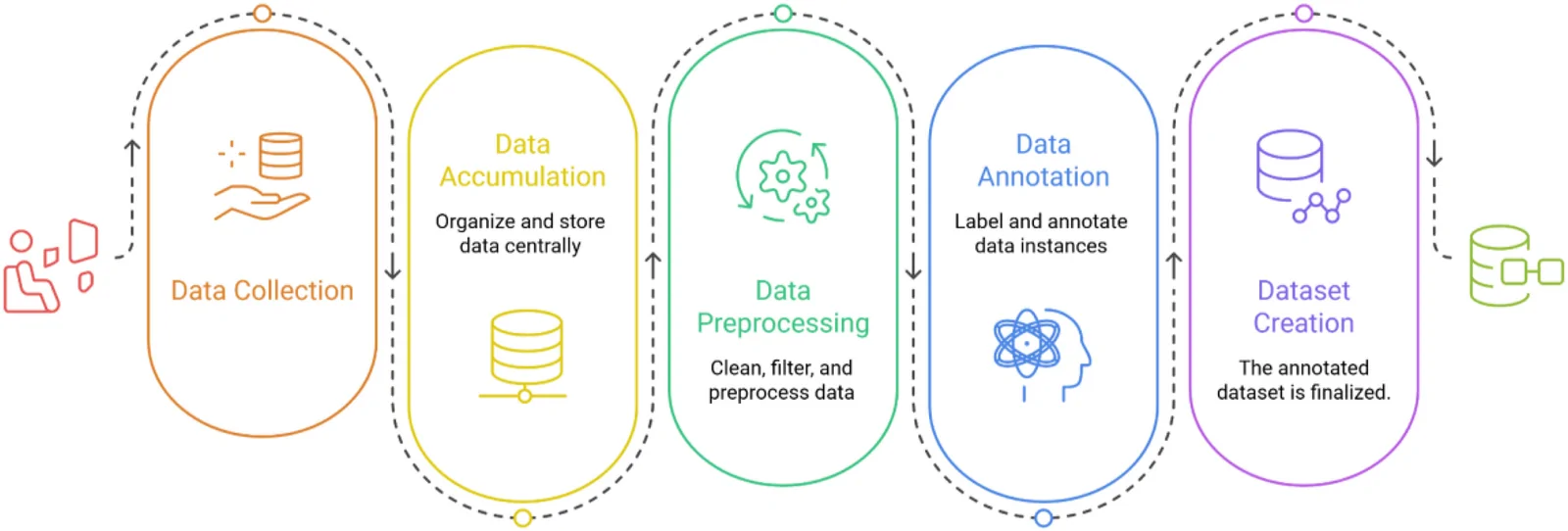
Dataset development workflow.

This section elaborates on the data acquisition process, dataset distribution, and annotation procedure. We also provide visualizations of the datasets, such as word clouds, and discuss some examples of disagreements in annotations that were encountered during the process of creating the datasets.

### 3.1 Dataset acquisition

The use of internet and social media has significantly expanded Bengali language communication in digital space in Bangladesh. DataReportal-2025^1^ shows that in 2025 Bangladesh has an estimated total of 77.7 million internet users and over 60 million social media user identities. These online spaces offer a wide range of opportunities for the viewing of current gender-related language, stereotypes, hostility and social attitudes.

To ensure that the dataset reflected naturally occurring linguistic and contextual patterns rather than relying solely on the authors’ assumptions, an exploratory review of publicly accessible Bengali online discourse was conducted prior to sample development. The review covered Facebook public pages and discussions, YouTube comments, Instagram public posts and comments, Twitter/X discussions, online news portals and their comment sections, and selected public blogs and forums. These sources were explored as they constitute a variety of informal Bengali communication and often include discussions about social, cultural, professional, and gender issues.

Special focus was placed on posts, videos, news reports, and discussion threads that were particularly engaging in terms of women and gender-related issues. These covered women's professional life, education, employment, leadership, social life, empowerment, relationship, marriage, domestic work, clothing and appearance, entertainment and sports, violence against women and sexual harassment. Such discussions often contained a wide range of responses, including supportive and neutral comments, stereotypical judgments, derogatory expressions, sexualized remarks, morality-based criticism, and gender-directed hostility.

The review focused on recurring linguistic and contextual patterns rather than collecting individual comments as dataset instances. Observed patterns included respectful and supportive statements, gender-role generalizations, derogatory labelling, morality-based criticism, negative judgments regarding women’s competence or behaviour, appearance-focused insults, sexualized remarks, objectification, and expressions that normalized or trivialized harassment.

The final 7,017 Bengali text samples were independently developed by the authors using the recurring linguistic structures, thematic contexts, and interaction patterns identified during the exploratory review. Particular focus was placed on variation in vocabulary, sentence structure, context, intensity, and communicative intent. The online discourse reviewed was used only as a contextual and linguistic reference and no particular social media post, comment, username, personal identifier, or directly traceable content was copied, reproduced, or directly paraphrased.

While the final set of dataset instances was developed by the authors, the language and themes were derived from the more frequently occurring expressions and interactions in publicly available Bengali online discourse. The exploratory review therefore provided a real-world reference framework for dataset construction while avoiding the reproduction of identifiable user-generated content.

Representative samples showing the relationship between the reviewed online discourses and the independently developed instances in the dataset are shown in Table 1.

**Table 1:** Illustrative relationship between reviewed online discourse patterns and dataset samples.

### 3.2 Dataset distribution

The dataset consists of 7,017 instances in four categories: Non-Misogynistic (2,420), Stereotype (1,744), Derogation (1,527), and Sexual Harassment (1,326). We retained a relatively larger Non-Misogynistic class to help the model learn clearer boundaries between misogynistic and non-misogynistic language. This distinction is necessary for the fine-grained and coarse-grained classification tasks in developing models with the corpus. Fig. 2 depicts the class-level distribution of samples in the dataset.

**Fig. 2:**
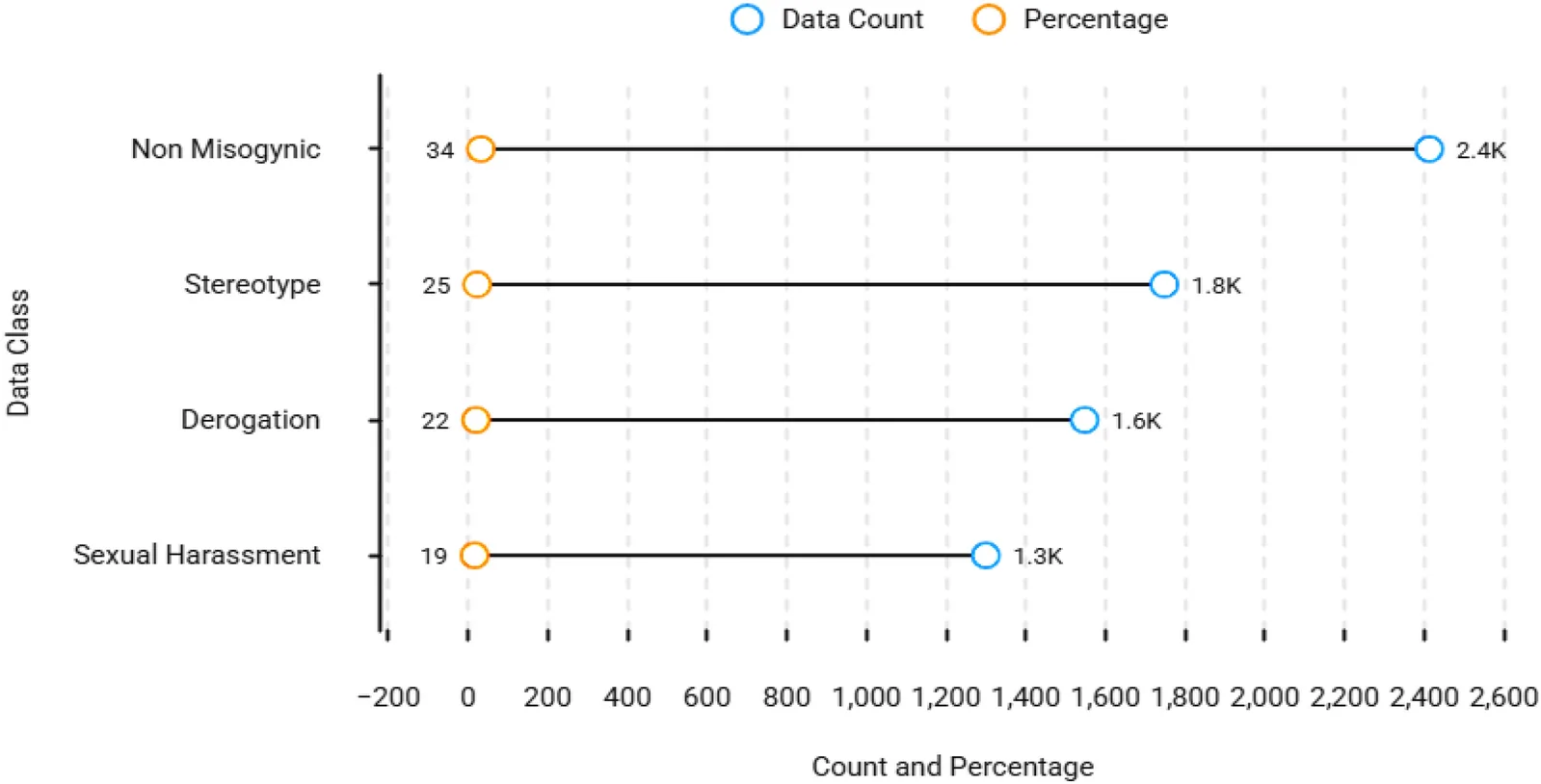
Analysis of dataset distribution with sample counts.

### 3.3 Annotation procedure

The annotation procedure was carefully designed to develop a high-quality and reliable labelled corpus that captures the nuanced forms of misogynistic expression in Bengali online discourse. For this task, a set of four trained annotators was recruited. The annotators were all native speakers of Bengali with good knowledge of Bengali sociolinguistic norms and online communication and cultural patterns. Each of the instances was annotated by two independently assigned annotators from a pool of four, based on availability and workload distribution. Those cases that had 100% annotator agreement were finalized and did not require additional review. In cases of disagreement, the instance was referred to a third annotator with domain expertise, whose adjudicated decision was adopted as the final authoritative label. A clear and structured set of annotation criteria was created to ensure consistency and decrease the subjectivity of the annotations. Since perceptions of misogynistic content may vary due to cultural background, personal experience and subjective interpretation, the criteria provided a common decision-making framework for all annotators. Furthermore, prior to beginning the annotation process, the annotators were given sample instances and associated labels to have a practical understanding of the guidelines for annotation. The systematic annotation criteria used in the annotation workflow are presented in Fig. 3.

**Fig. 3:**
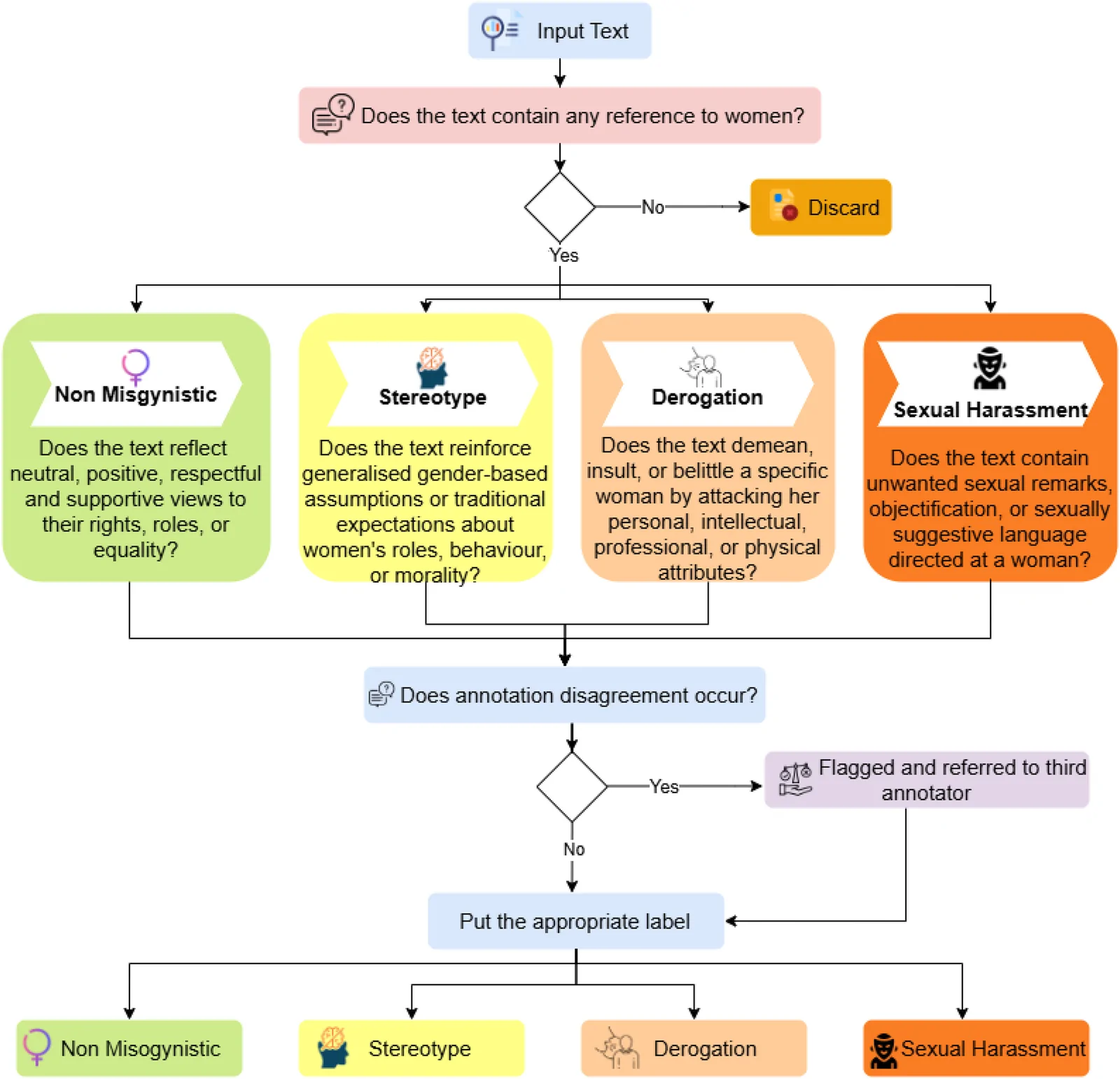
Dataset annotation guidelines.

To assure accurate and consistent annotation, it is crucial to maintain clear definitional boundaries among classes and preserve their mutual exclusiveness through a well-structured annotation workflow. The complete annotation procedure is formalized in Algorithm 1.

**Algorithm 1:** Stepwise procedure for data annotation.

**Input**: Set of preprocessed text instances
**Output:** Annotated misogyny detection dataset

Phase 1: Independent Dual Annotation

1. T ← {t1, t2, . . ., tn} (set of text instances) 2. OMANISHA ← [] (final annotated dataset) 3. L ← {Non Misogynistic, Stereotype, Derogation, Sexual Harassment}
4. A ← {A1, A2, A3, A4} (pool of four annotators) 5. D_disagree ← []
6. for ti ∈ T do 7. {Ap, Aq} ← randomly select 2 annotators from A (p ≠ q) 8. 9. l1 ← Ap assigns label to ti ⊳ l1 ∈ L ∪ {flag} 10. l2 ← Aq assigns label to ti ⊳ l2 ∈ L ∪ {flag} 11. 12. if l1 == flag and l2 == flag then 13. Discard ti 14. else if l1 == l2 then 15. OMANISHA.append(ti, l1) 16. else 17. D_disagree.append(ti, l1, l2) 18. end if

Phase 2: Expert Adjudication of Conflicted Instances

19. for dj ∈ D_disagree do
20. if lf == discard then 21. Discard dj 22. else OMANISHA.append(dj, lf) 23. end if 24. j ← j + 1 25. end for

Phase 3: Quality Validation

26. κ ← Cohen's Kappa(A labels) 27. α ← Krippendorff's Alpha(A labels) 28. if κ ≥ 0.60 and α ≥ 0.60 then 29. Accept OMANISHA as final dataset 30. else 31. Revise annotation guidelines 32. Repeat Phase 1 33. end if
34. return OMANISHA

The academic and professional qualifications of the third expert annotator assigned to resolve annotation disagreements are presented in Table 2.

**Table 2:** Academic and professional qualifications of the third expert annotator.

Attribute	Third Expert Annotator
**Academic qualification**	PhD
**Domain expertise**	Natural Language Processing (NLP), text classification, and harmful-content analysis
**Research experience**	Extensive research experience in NLP, machine learning, and analysis of online textual content
**Years of experience**	15 years
**Technical expertise**	Expertise in linguistic and contextual interpretation of Bengali text, annotation, and classification of implicit and explicit harmful expressions
**Mother language**	Bengali

### 3.4 Calculation of the annotators agreement

The measurement of annotator agreement is an essential process in creating a reliable annotated dataset. In the case of the OMANISHA dataset, the inter-annotator agreement was systematically assessed by two common methods: Cohen's kappa (κ) and Krippendorff's alpha (α). These measures evaluate complementary aspects of annotation consistency and offer a holistic assessment of the quality of the annotations in the dataset.

#### 3.4.1 Cohen's Kappa (κ)

Cohen’s Kappa evaluates inter-annotator reliability by measuring the agreement between two annotators while accounting for agreement that may occur by chance. For a given pair of annotators, Cohen's Kappa is formally defined as:

(1) $$\kappa \;=\frac{\;P\;o\;-\;P\;e}{1\;-\;P\;e}$$

In this context, Po represents the observed agreement between the annotators, while Pe refers to the agreement expected to occur randomly.

Table 3 reports the Cohen’s Kappa values, with the strongest agreement observed for the non-misogynistic class (0.82) and the weakest agreement recorded for the derogation class (0.71). The overall average kappa score of 0.76 indicates substantial agreement among the annotators.

**Table 3:** Cohen’s Kappa Scores by Class.

Class Name	Kappa Score	Mean Score
Non Misgynistic	0.82	0.76
Stereotype	0.75	
Derogation	0.71	
Sexual Harassment	0.76	

#### 3.4.2 Krippendorff's Alpha (α)

Krippendorff’s Alpha provides a versatile measure of annotation reliability, allowing agreement assessment across multiple annotators and various measurement scales. Krippendorff's Alpha is formally defined as:

(2) $$\alpha \;=1-\;\frac{\;\mathrm{Do}\;}{\mathrm{De}}$$

Here, Do denotes the observed disagreement recorded across all annotator pairs, and De denotes the expected disagreement by chance.

Table 4 reports the Krippendorff’s Alpha values, with the highest reliability observed for the non-misogynistic class (0.83) and the lowest reliability recorded for the derogation class (0.70). The average alpha score of 0.75 indicates a strong level of annotation consistency.

**Table 4:** Krippendorff's Alpha value for each class.

Class Name	Kappa Score	Mean Score
Non Misogynistic	0.83	0.75
Stereotype	0.72	
Derogation	0.70	
Sexual Harassment	0.76	

### 3.5 Examples of annotation disagreements

Table 5 illustrates selected cases of inter-annotator disagreement, wherein conflicting labels were adjudicated through domain expert consultation to arrive at the final classification.

**Table 5:** Examples of annotation discrepancies with expert feedback.

### 3.6 Dataset sample

A set of representative samples from the OMANISHA dataset and their corresponding final labels are shown in Table 6.

**Table 6:** Sample texts with corresponding categories in the OMANISHA dataset.

### 3.7 Dataset visualization

Word clouds were created for the four annotated classes in both original Bengali and English translated data to give an intuitive and comprehensive idea about the linguistic characteristics and structural composition of the data. Each word cloud highlights the most frequently occurring and lexically distinctive terms within its respective class. Fig. 4 shows the word clouds for the original Bengali data, while Fig. 5 shows the word clouds for the English translated data.

**Fig. 4:**
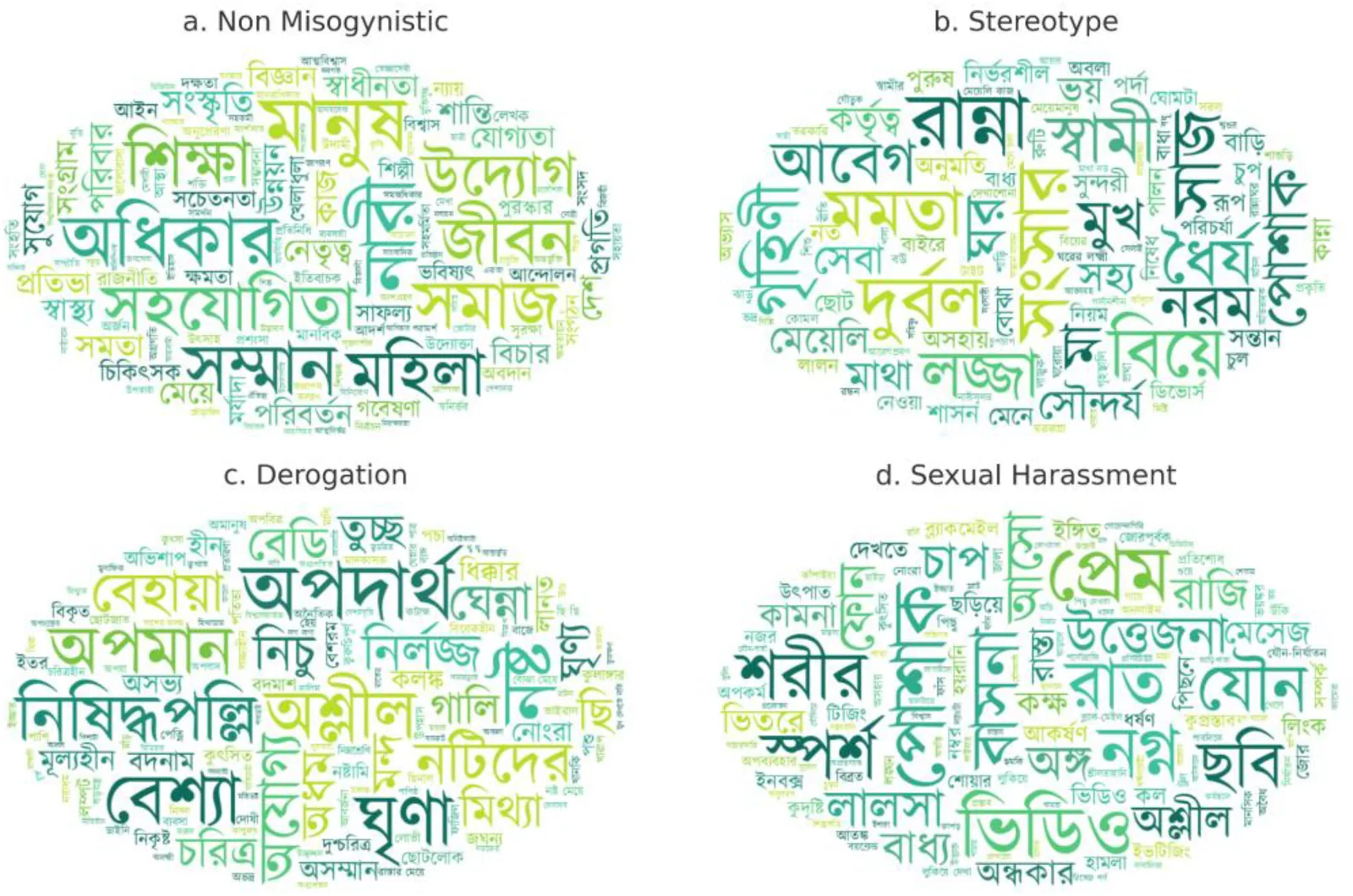
Class-wise word clouds of the Bengali dataset.

**Fig. 5:**
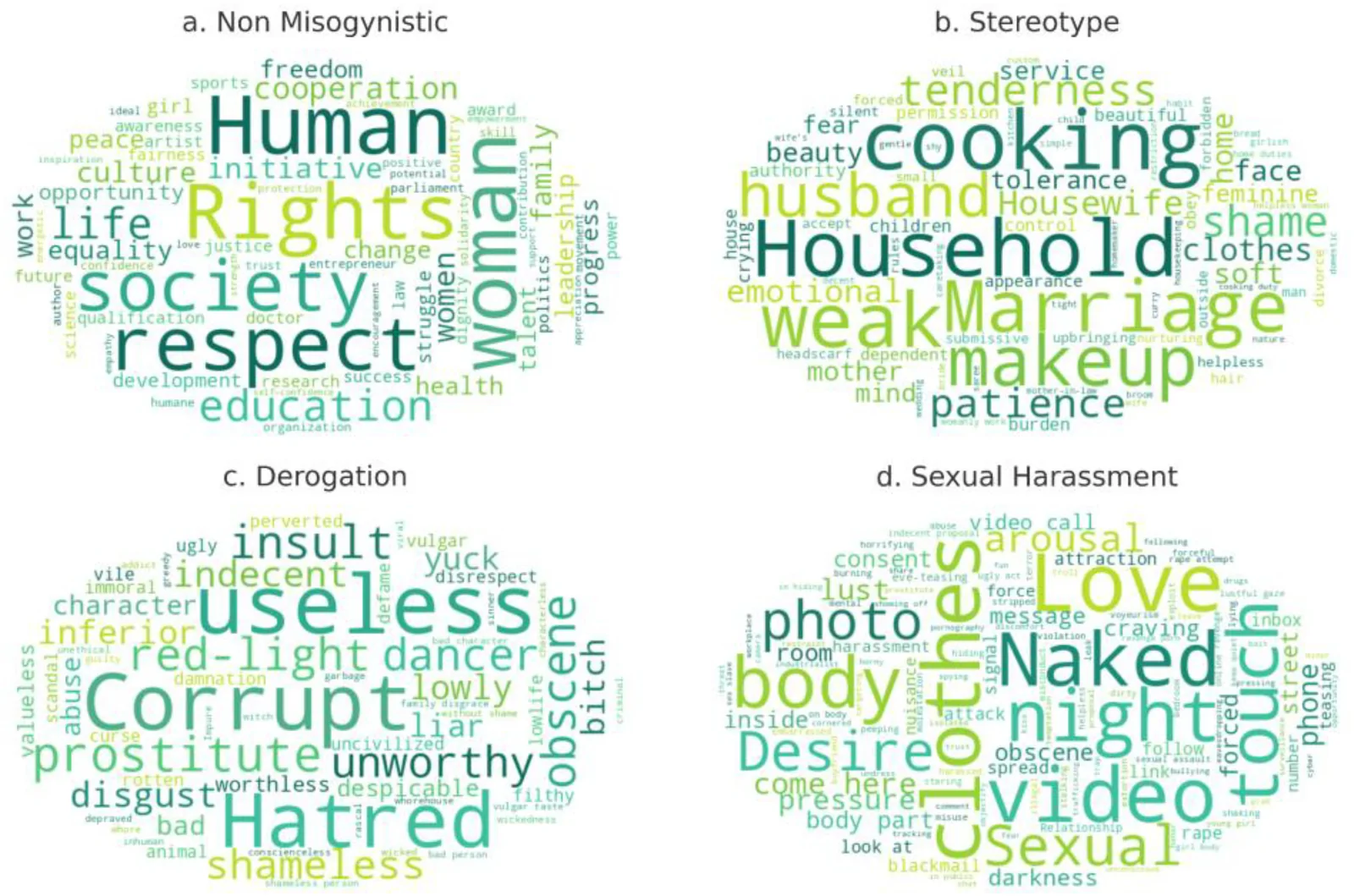
Class-wise word clouds of the English-translated dataset.

## 4 Experimental Design, Materials and Methods

This section presents the data preprocessing procedures, dataset development workflow, statistical analysis of the dataset and class-wise lexical overlap analysis.

### 4.1 Preprocessing

Raw text data is often noisy, unstructured and inconsistent, and must undergo a rigorous preprocessing pipeline to guarantee linguistic integrity before annotation. Therefore, all instances were systematically preprocessed to remove noise and improve linguistic consistency. Duplicate entries were eliminated and hashtags, markup, and other formatting artifacts were removed. Excessive whitespace, repeated punctuation, and irregular spacing were normalized. Code-mixed cases were kept if there was a clear communicative intent in Bengali. Finally, instances below the minimum token threshold were removed, leaving a cleaned corpus of 7,017 instances that were then passed on to the annotation phase. The minimum token threshold for our dataset is three which was selected to remove extremely short or fragmentary samples with insufficient linguistic context while retaining short but meaningful expressions relevant to Bengali misogyny detection.

### 4.2 Dataset development workflow

A systematic annotation process was applied to the preprocessed dataset, where each instance was classified into one of the four predefined and mutually exclusive classes according to structured annotation guidelines. Four trained native speakers of Bengali, who were familiar with Bengali sociolinguistic norms and online communication patterns, were involved in the annotation process. Each instance was independently labeled by two annotators assigned from a four-member annotation team based on their availability and workload balance. If both annotators assigned the same label, then the label was accepted as the final consensus label. Where there was a disagreement, the instance was taken to a third annotator who has an expertise in the domain, and his verdict was taken as the authoritative label. Cohen's Kappa (κ = 0.76) and Krippendorff's Alpha (α = 0.75) were used to assess the reliability of the annotation process, which resulted in substantial and acceptable inter-annotator agreement. The entire process that was used in the development of the dataset is shown in Fig. 6, spanning from preliminary data collection to the determination of the final labels.

**Fig. 6:**
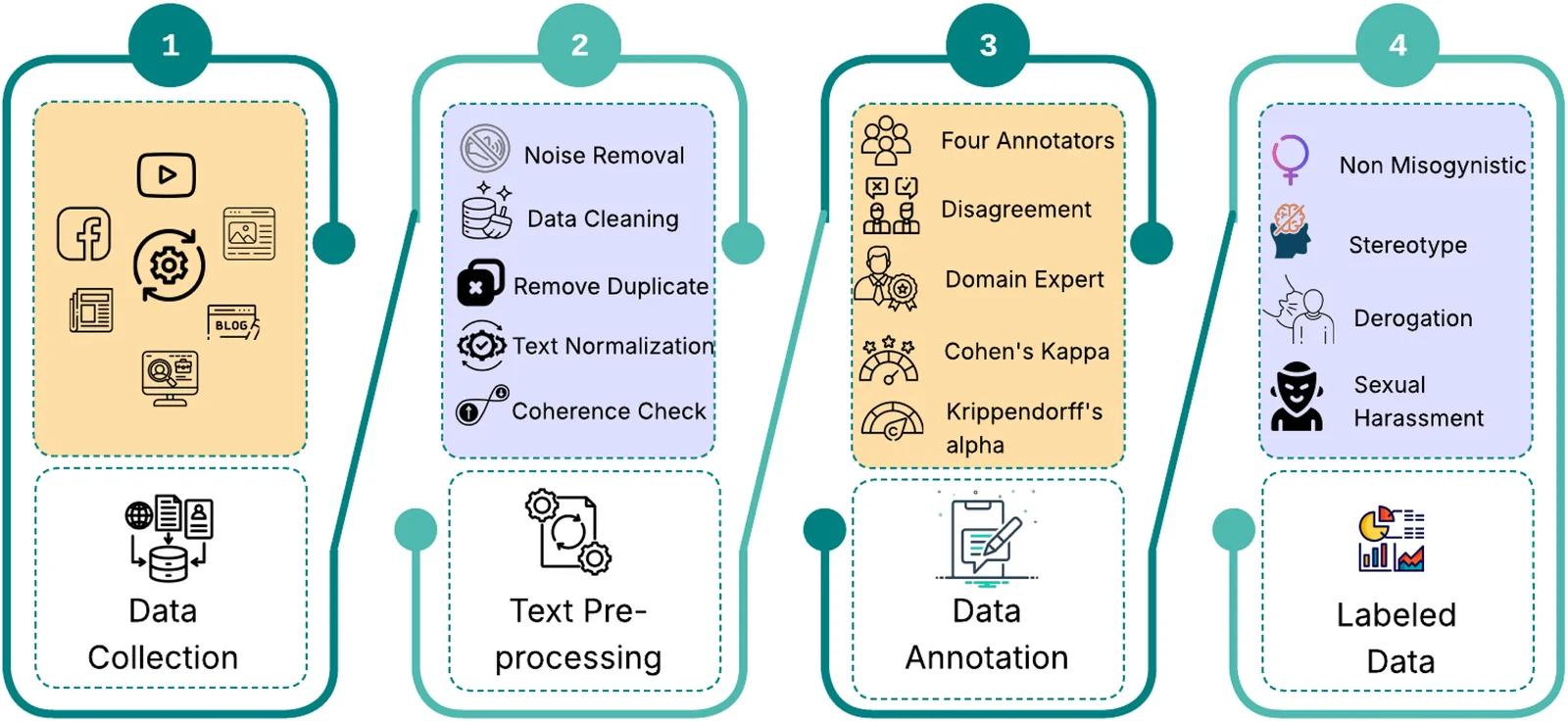
Schematic Overview of the Dataset Development Process.

### 4.3 Dataset analysis

Key statistical measurements were calculated across the four classes of the OMANISHA dataset and are shown in Table 7 and Fig. 7 to characterize the linguistic composition of the dataset. The dataset includes a total of 7,017 instances and 59,008 words with a Maximum Text Length (MTL) of 26 words and Average Number of Words per Text (ANW) of 8.41, which shows that the texts are predominantly short to medium length.

**Table 7:** Summary of Text Statistics Across Classes.

Class Name	Total words	MTL (words)	ANW (per text)	Unique words	ANUW (per text)	TTR
Non Misgynistic	18330	17	7.57	3350	7.50	0.18
Stereotype	16136	26	9.25	2472	9.04	0.15
Derogation	13036	21	8.54	2451	8.43	0.19
Sexual Harassment	11506	23	8.68	1716	8.51	0.15
Overall	59008	26	8.41	6429	8.27	0.11

(ANW = Average Number of Words per Text, MTL = Maximum Text Length, TTR = Type-Token Ratio, ANUW = Average Number of Unique Words per Text).

**Fig. 7:**
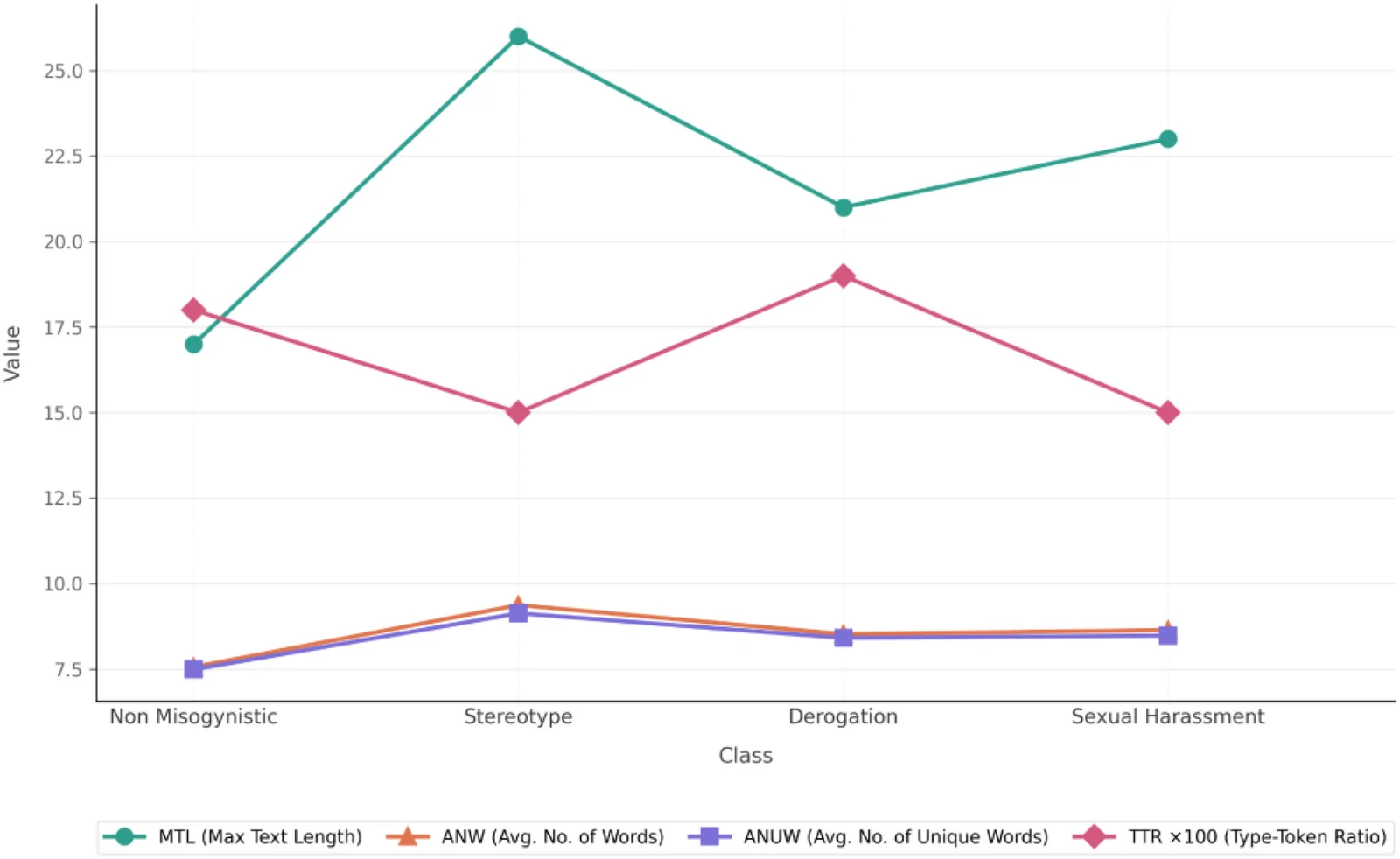
Comparison of textual statistics across dataset classes.

At the class level, the Non-Misogynistic category has the highest total word count (18,330) and shows relatively high lexical diversity, with a Type-Token Ratio (TTR) of 0.18, as it covers varied neutral expressions. The Stereotype class has the highest MTL (26), ANW (9.25), and Average Number of Unique Words per Text (ANUW) (9.04), indicating that stereotypical comments tend to be longer and more descriptive, as they are usually general statements regarding women's roles, behavior, or social expectations. The Sexual Harassment class exhibits the lowest word count (11,506) and lower TTR (0.15), which indicates that explicit terms are used less frequently and more repetitively. Derogation, by contrast, is the most extreme in terms of TTR (0.19), which reflects a greater range of abusive and insulting vocabulary. In general, the differences are due to the different communicative patterns and lexical choices each class has.

Table 8 reports the distribution of text length frequencies of each class of the dataset and Fig. 8 reports an analytical summary of text length frequencies for different classes of the dataset. The analysis reveals that the majority of occurrences are within the medium-length range and the highest frequencies are between 6 and 10 tokens. This indicates that the dataset is mainly composed of short to moderately sized instances. The Non-Misogynistic class has the shortest texts, with a mean length of 7.58 tokens. The Stereotype class, on the other hand, has comparatively longer texts, with the highest mean length of 9.32 tokens and the highest length of 26 tokens. Derogation and Sexual Harassment classes exhibit intermediate behaviour with an average of 8.55 and 8.69 tokens, respectively. Nevertheless, the standard deviations for all the classes are relatively low, reflecting similar distributions of text lengths, which suggests that the corpus is dominated by short expressions common in modern Bengali online discourse.

**Table 8:** Text length distribution for each class.

Class Name	Count	Mean	Std	Min	25%	50%	75%	Max
**Non-Misogynistic**	2420	7.58	2.38	3	6.00	7.00	9.00	17
**Stereotype**	1744	9.32	2.89	3	7.00	9.00	11.00	26
**Derogation**	1527	8.55	2.28	3	7.00	8.00	10.00	21
**Sexual Harassment**	1326	8.69	2.45	3	7.00	8.00	10.00	23

**Fig. 8:**
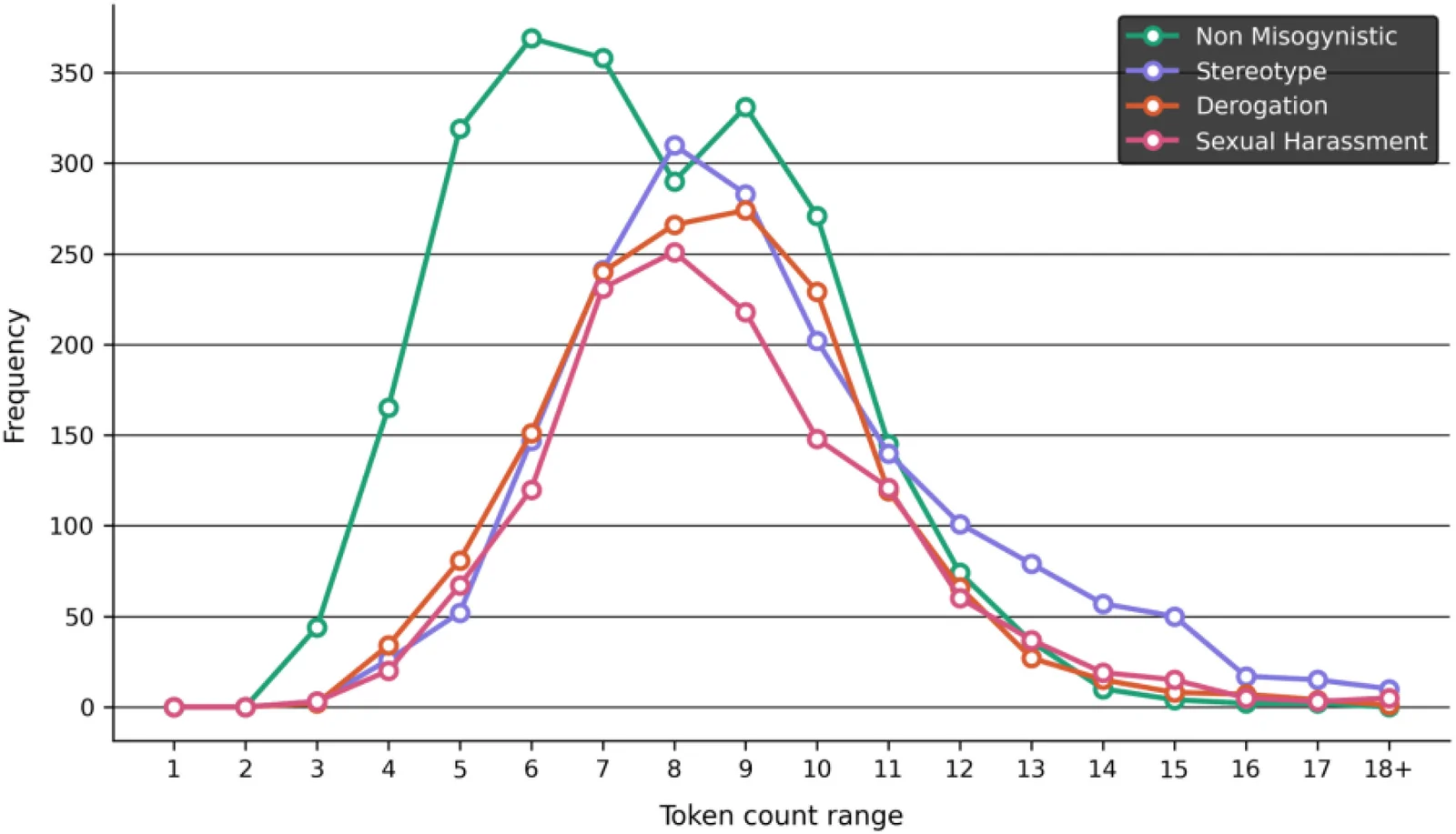
Token frequency count per class.

### 4.4 Class-wise lexical overlap analysis

The lexical overlap for classes is shown in Fig. 9 measured using Jaccard similarity. The low values of the off diagonal scores (0.12 to 0.21) suggest that the classes have separable lexical features. Stereotype and Derogation have the greatest similarity (0.21), indicating that there may be some overlap in negative or gendered expressions. The lowest similarity, on the other hand, between Non-Misogynistic and Sexual Harassment (0.12) shows a distinct lexical distinction between neutral and explicitly sexualized terms.

**Fig. 9:**
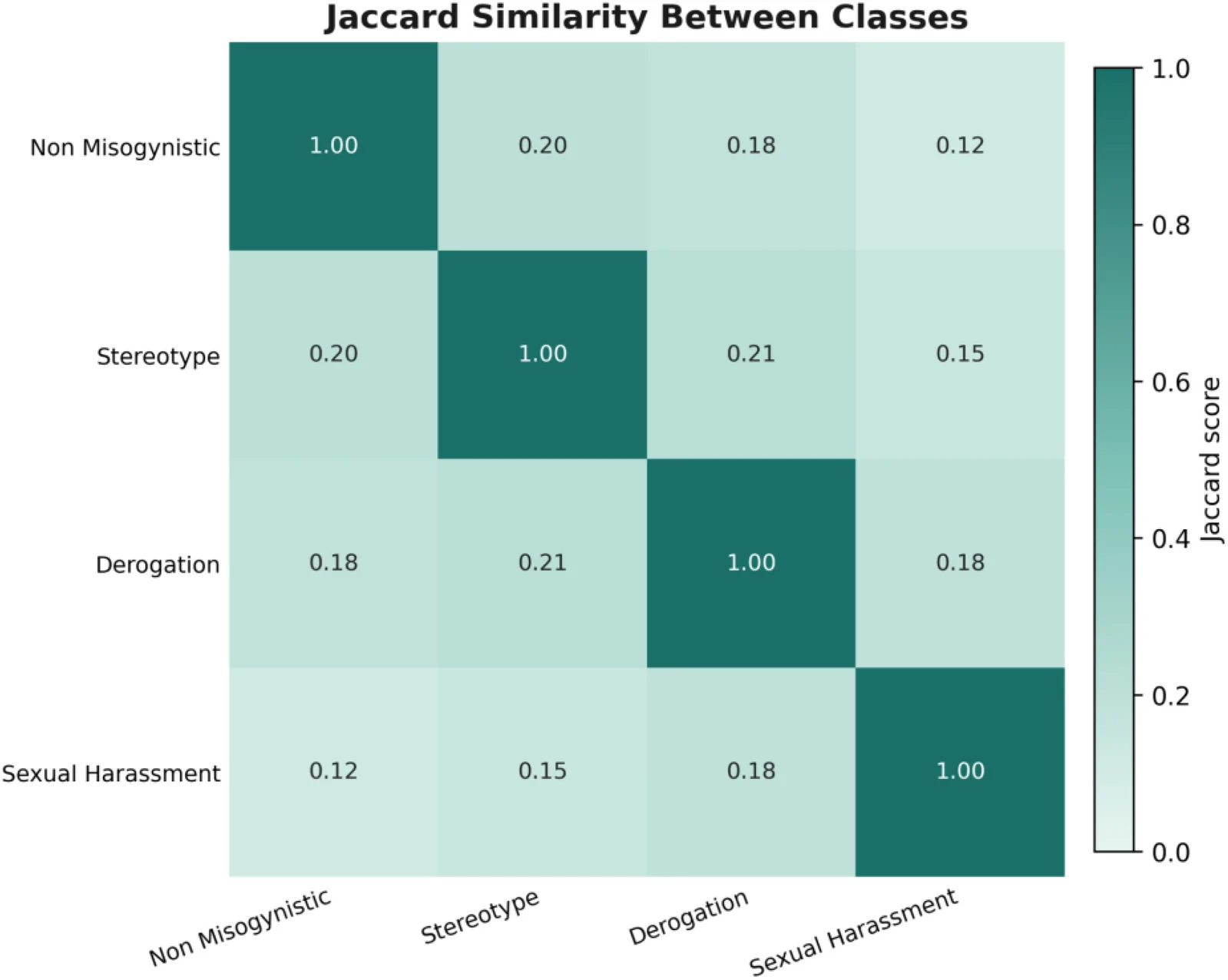
Jaccard similarity value for each class.

## Limitations

While the OMANISHA data set can serve as a valuable resource for misogynistic text identification in the Bengali online discourse, it has few limitations. Future extensions may include more nuanced forms of expression, such as implicit hostility, sarcasm, and contradictory sentiment, to strengthen the dataset’s overall reliability and coverage. Also, future versions will contain more code-mixed and code-switched Bengali-English-Hindi examples to enhance robustness and real world generalizability.

## Ethics Statement

The OMANISHA dataset was developed in accordance with responsible research and ethical principles. Public online discourse was reviewed only to understand relevant linguistic patterns and contextual features, while all samples were independently created and annotated by the authors without copying or directly paraphrasing existing content. The dataset was further screened to minimize bias, harmful framing, privacy concerns and unfair targeting of any individual, community, religion, or social group. The main purpose of this dataset is to assist in developing reliable content moderation tools for limiting online misogynistic abuse, rather than promoting criticism, disrespect, or hostility toward any belief system, community, or individual group. The dataset is intended strictly for responsible research, educational and benchmarking purposes, particularly for the development and evaluation of automated misogyny detection systems. No personal or sensitive information relating to identifiable individuals is included in the dataset. Given the potentially harmful nature of misogynistic language, users are strongly discouraged from using the dataset to generate, reproduce, or disseminate abusive content, or to target, harass, or discriminate against any individual or group in ways that may cause harm.

## Credit Author Statement

**Fatama Jannat Tisha:** Conceptualization, Data curation, Methodology, Software, Writing – original draft, Visualization. **Bibhas Roy Chowdhury Piyas:** Conceptualization, Data curation, Methodology, Software, Writing – original draft, Visualization. **Nurjahan Afrose:** Data curation, Methodology, Investigation, Validation, Writing – review & editing. **Sabbir Ahmed:** Data curation, Methodology, Investigation, Validation, Writing – review & editing. **Shahrin Islam:** Data curation, Methodology, Investigation, Validation, Writing – review & editing. **Shazzad Hossen:** Methodology, Investigation, Validation, Writing – review & editing. **Jafrin Iqbal Chowdhury:** Methodology, Investigation, Validation, Writing – review & editing. **Afsana Begum:** Methodology, Investigation, Validation, Writing – review & editing. **Imran Mahmud:** Methodology, Investigation, Validation, Writing – review & editing

## Funding Statement

This research was supported by Daffodil International University, Bangladesh.

## Data Availability

Mendeley DataOMANISHA: A Benchmark Dataset for Identifying and Categorizing Bengali Misogynistic Text (Original data)

## Footnotes

1 https://datareportal.com/reports/digital-2025-bangladesh
